# Simultaneous Multibeam Clustered Phased Arrays Analysis Using Mixed and Multiple Antenna Element Factors

**DOI:** 10.3390/s24237801

**Published:** 2024-12-05

**Authors:** Francesco Alessio Dicandia, Simone Genovesi

**Affiliations:** 1 Istituto di Elettronica e di Ingegneria dell’Informazione e delle Telecomunicazioni, Consiglio Nazionale delle Ricerche, 56122 Pisa, Italy; 2 Dipartimento di Ingegneria dell’Informazione, Università di Pisa, 56122 Pisa, Italy; simone.genovesi@unipi.it

**Keywords:** multibeam, phased array, Penrose subarrays, Pareto optimization, wide-angle scan

## Abstract

A novel design strategy for improving the radiative performance of simultaneous multibeam (SMB) phased arrays is addressed. The proposed scheme relies on the adoption of mixed and multiple antenna element factors with a dynamic selection of their radiation patterns whose choice depends on the desired SMB pointing directions. In addition, a Penrose-inspired clustering technique is also employed for reducing the array feed points. Compared with traditional phased arrays based on a single antenna element factor, the novel array architecture allows the scan angle range to be widened by improving the minimum array gain as well as reducing the peak side lobe level (PSLL). The superior radiative performance of the proposed approach with respect to the clustered phased arrays with a single-mode element factor is assessed in SMB scenarios comprising two and three main lobe peaks. The notable SMB radiative improvement has been also confirmed from a statistical point of view by considering up to four and five concurrent main lobes. The remarkable radiative improvements confirm the effectiveness of the proposed solution, which also represents an appealing candidate for its exploitation in multiuser and multibeam communications.

## 1. Introduction

The emerging wireless applications and services related to beyond-5G and -6G systems, along with the Internet of Things (IoT) paradigm, are increasingly prompting the next wireless communications toward millimeter-wave (mmWave) spectrum or even higher carrier frequencies [1]. Indeed, this frequency range allows reliable communication networks to be supported with the expected quality of service and deals with the pressing need for large channel bandwidths. However, differently from their sub-6 GHz counterparts, mmWave communications are prone to many impairments, such as a more pronounced power spreading as well as a low power-amplifier efficiency, especially if affordable and power-efficient hardware components are considered [2]. Therefore, to mitigate these undesired drawbacks, high-gain antennas offering a directional beam represent a promising key technology that can be employed on both the transmitter and the receiver sides using a large number of radiating elements in a compact physical space. By this means, recent advancements in wireless communication systems have fostered the research and development of novel phased array transceivers [3]. To date, several phased arrays have been proposed for different mmWave applications [4,5,6]. Dual polarized phased array transceivers have been also designed [7] to provide two concurrent independent beams capable of doubling channel capacity. An interference reduction method based on stacking multiple uniform linear phased arrays has also been presented [8]. The design and experimental verification of a phased array based on gap waveguide technology has been proposed [9]. Meanwhile, the spectral efficiency improvement of triangular lattice phased array has been proved [10].

It is interesting to note that, while these solutions are effective in the case of point-to-point (PtP) links, they could be extremely inefficient in case of simultaneous multibeam (SMB) scenarios such as massive multiple-input-multiple-output (MIMO) [11] or multicast mode traffic delivery [12], where the same data must be delivered to multiple users or devices. In addition to terrestrial infrastructures, the need for SMB for point-to-multipoint (PtM) links can also be advantageously exploited in the framework of space–air–ground integrated networks [13], where low-earth-orbit (LEO) satellites [14] or high-altitude platform stations (HAPS) [15] are employed to improve both communication quality and widen the beam coverage for non-terrestrial links.

There are several technological implementations of SMB antennas that exhibit different complexity, flexibility, and cost. Multibeam phased array antenna technology has some pivotal advantages with respect to solutions that rely on lenses [16], engineered panels [17,18,19], or passive beamforming circuits [20] due to its capability to provide steerable SMB by using a proper beamforming (BF) strategy and precoding weights for serving various users within a predefined angular sector [21,22]. This is a crucial feature that can guarantee the necessary wireless connectivity level even in cases of rapid changes in the position of a ground user or a non-terrestrial platform. However, most of the listed technology solutions are focused on a single steerable beam, while the SMB phased array (SMBPA) synthesis has gathered little attention [23,24,25,26].

The purpose of this work is to propose a novel array architecture aiming to improve SMBPA radiative performance. The envisioned approach exploits a Penrose-inspired clustering array technique that operates on the regular lattice of the radiating elements [26,27] and introduces the use of multiple and dynamic antenna element factors, allowing the minimum gain value to be enhanced, as well as lowering the peak side lobe level (PSLL), with respect to an SMBPA that adopts only a single-antenna element factor. Although a mixed antenna element factor exhibiting a static radiation pattern (RP) has been proposed for a single-beam scenario [27], the choice of using mixed and multiple antenna element factors with a dynamic selection of their RPs is exploited for the first time to the best of the authors’ knowledge.

The rest of the article is organized as follows. Section 2 describes the design approach based on the Penrose tessellation and the exploitation of multiple element factors with dynamic RP for determining the suitable SMBPA partitions. The performance enhancement offered by the proposed SMBPA array architecture with respect to one that adopts a single-mode element factor is reported in Section 3 in the cases of two and three main lobe peaks. Section 4 is devoted to the statistical analysis in the cases of SMB with four and five main beams. Finally, the conclusions are drawn in Section 5.

## 2. Problem Formulation of the SMBPA with Multiple Antenna Element Factors

The RP of an SMBPA, lying on the *xy* plane and partitioned into *Q* tiles, can be evaluated by applying the superposition principle to the whole array panel as follows:

(1) $$P\left(u,v\right)=\sum_{b=1}^{B}\left(\sum_{q=1}^{Q}a_{q,b}\sum_{n=1}^{N_{q}}E_{q,n}\left(u,v\right)e^{j\beta \left(x_{q}u+y_{q}v\right)}\right)$$

where *u* = sin(*θ*)cos(*ϕ*), *v* = sin(*θ*)sin(*ϕ*), *β* = 2π/λ, λ is the wavelength at the working frequency, *N_q_* is the number of radiating elements of the *q^th^* tile, *E_q_*_,*n*_(*u*, *v*) represents the *n^th^* element radiation pattern within the *q^th^* tile with centroid equal to (*x_q_*, *y_q_*), *a_q_*_,*b*_ stands for the complex precoding coefficients, and *B* expresses the number of main lobe peaks. In an SMBPA, inter-beam interference plays a crucial role in determining the signal-to-interference-plus-noise ratio (SINR) [8]. Although various precoding schemes [28], such as zero forcing, can suppress beam interference, they rely on both accurate channel knowledge and antenna calibration, which is practically not realistic without significant signal overhead. Therefore, the maximum ratio (MR) linear precoding strategy was adopted since it can be considered more reliable in most SMB scenarios. It is worth observing that in the proposed design approach, the kind of selected BF strategy does not play a primary role. Indeed, the complex precoding coefficients applied to the *Q* tiles can be implemented with any BF method (e.g., analog, digital, or hybrid), each one with known performance limitations [20]. For instance, in the case of analog BF, the SMB via phase shifters and power amplifiers provides the same stream of data, which can be dedicated to a single user or a different group of users in the case of multicast delivery. Moreover, a digital or hybrid BF scheme [22] may be used to distinguish the streams of data associated with each main lobe peak, thus realizing spatial multiplexing.

### 2.1. SMB Scenario

**Figure 1:**
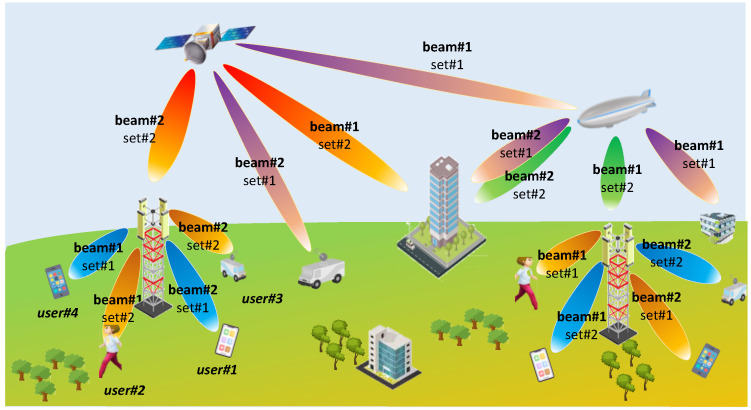
Example of SMB base station antennas that have a link with two users at a time but that can also select the beam directions based on a set of predefined directions.

An example of the envisioned operative scenario for the novel phased array architecture is illustrated in Figure 1. It comprises two master stations each one exploiting an SMB antenna RP to deliver data to multiple users simultaneously.

In Figure 1, the SMB base station antennas establish a link with two users at a time and can also change the beam directions to follow the users. A rectangular sector cell in the *uv* plane with an elevation and azimuth angle of ±20° and ±60°, respectively, has been considered to assess the performance improvement of the proposed SMBPA based on a multiple antenna element factor. A set of SMB configurations with two and three simultaneous main beams has been considered to investigate the most suitable clustering of a periodic phased array as well as to study the benefits of the multiple antenna element factor selection. Without loss of generality, the selected beam directions comprise the broadside (*θ* = 0°, *ϕ* = 0°) and the combinations (*θ* = 60°, *ϕ* = ±20°) and (*θ* = 60° and *ϕ* = ±160°). These five directions define ten different SMB configurations each with two or three main lobes. It is worth noting that the selected five pointing directions are used just as an example and different or additional pointing directions could be considered during the phased array synthesis, according to the operative SMB scenario as well as the system requirements.

### 2.2. Multiple Antenna Element Factors

**Figure 2:**
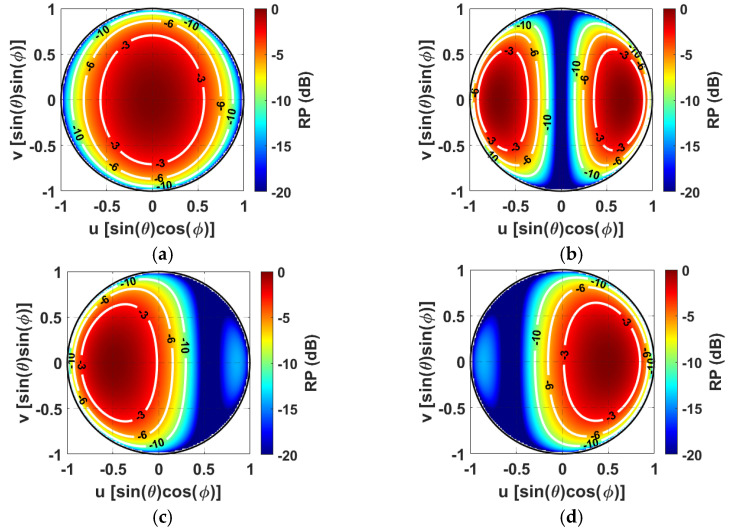
Available set of normalized element patterns in the *uv* plane; (**a**) ∆*φ* = 0° (*EF_1_*), (**b**) ∆*φ* = 180° (*EF_2_*), (**c**) ∆*φ* = 120° (*EF_3_*), and (**d**) ∆*φ* = −120° (*EF_4_*).

Since a microstrip patch antenna radiates as an array of two magnetic dipoles [29], the set of available RPs is derived by considering these radiators separated by a distance *L* representing the patch length. Thereby, by introducing a phase delay (∆*φ*) between the two radiators lying on the *y*-axis, the corresponding element pattern (*EF*), turns out to be:

(2) $$EF\left(u,v,\Delta \varphi \right)=F\left(u,v\right)\left[2\cos\left(\frac{\beta Lv+\Delta \varphi }{2}\right)\right]$$

where *F*(*u*, *v*) stands for the element factor of the two magnetic dipoles. Figure 2 shows the normalized *EF* for four different phase delays (∆*φ*) with *F*(*u*, *v*) = cos(*θ*) and *L* = 0.3 λ. Specifically, the considered model enables the synthesis of radiating elements supporting either a patch-like (*EF_1_*), monopole-like (*EF_2_*), left-tilted patch (*EF_3_*), or a right-tilted patch (*EF_4_*) antenna array element with a ∆*φ* of 0°, 180°, 120° and −120°, respectively.

The addressed theoretical study introduces a novel SMBPA architecture merging the benefit of multiple-mode and dynamic element factors, differently from [27], where the selection between two static element patterns was optimized regardless of the single main lobe pointing direction. Specifically, the individual array elements can be dynamically selected among the *EFs* shown in Figure 2 according to the SMB pointing directions. It is worthy of note that the novelty addressed in this article does not lie in the four alternative RP shapes shown in Figure 2. Indeed, they are used just as a proof of concept to emphasize the potential SMBPA radiative performance based on multiple antenna element factors. Hence, according to the operative scenarios and the required system needs, different choices of antenna *EFs* could be employed [30,31]. Some examples of antenna design with radiated field shapes similar to the adopted ones are reported in [32,33,34].

### 2.3. SMBPA Design Method

In the proposed SMBPA design strategy, the periodic arrangement of the radiating elements has been organized into irregular and contiguous subarrays by exploiting an aperiodic Penrose tiling tessellation capable of covering a plane with a deterministic policy [27,35].

Specifically, the algorithm used for the synthesis of the proposed SMBPA architecture with mixed, multiple, and dynamic antenna element factors is generated through a recursive procedure as follows [35]:

1. Ensure there is an overlap between the regular and periodic phased array lattice and the Penrose tessellation vertexes;
2. Create a list with all the antenna elements of the periodic array that can be used for the phased array partition. At the beginning, the list contains all the antenna elements of the array;
3. Generate binary variables *x_n_* ϵ{0,1}, where *x_n_* (*n* = 1,…, *N*) and *N* is the total number of vertexes of the tessellation;
4. Call a recursive partition function for all the selected vertex *x_n_* = 1, starting from the first one in the list:
5. Define a polygon by merging all the isosceles triangles of the tessellation that have in common the picked vertex;
6. Ensure all the antenna elements of the periodic array inside the defined polygon form a subarray;
7. Remove the selected antenna elements of the periodic array from the list of available antenna elements for the phased array partition.
8. Assign a subarray with a single element for each antenna element that remains in the list of the available antenna elements;
9. Generate binary variables *y_m_* ϵ{0, 1} that define the RP of the array radiating elements, where *y_m_* (*m* = 1,…, *K*) and *K* is the total number of the array antenna elements.

It is worthy of note that the binary form of *y_m_* implies that in the addressed theoretical study, a selection policy that allows the exploitations of a maximum of two different antenna element factors for each SMB pointing direction represents the only constraint concerning the investigated SMBPA. To clarify this point, Table 1 describes the antenna element factor sets that are selected for the SMB configurations with two and three main beams.

**Figure 3:**
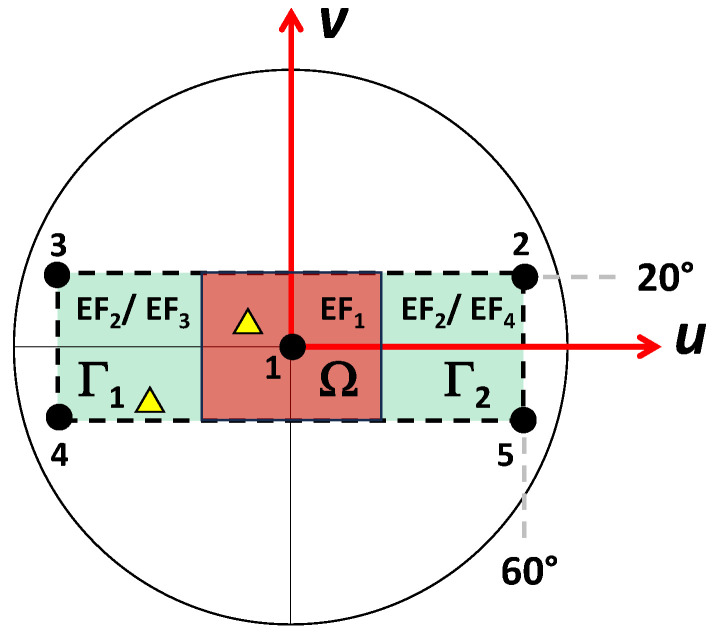
Rectangular scanning area with the five predefined SMB directions (black dots) and the three zones (Ω, Γ_1_, Γ_2_). Yellow triangle markers represent an example of a dual-beam steerable SMB pointing direction.

For example, since in the case of Conf#2.1, one beam is pointing broadside and the other toward the right hemisphere, the synthesis algorithm exploits this a priori knowledge by restricting the selection of the element pattern within the set comprising only element factors equal to *EF_1_* and *EF_4_*. Similarly, for Conf#2.2, the set includes only *EF_1_* and *EF_3_*. In the case of three simultaneous main lobes, the antenna element factors of the SMBPA are split into two subsets; the former radiating elements group is allowed to provide an element factor equal to *EF_1_* or *EF_2_*, and the latter *EF_2_*, *EF_3_*_,_ or *EF_4_*. It is worth noting that although the SMBPA is optimized by considering five fixed pointing directions, a steerable SMB can be achieved. Therefore, in general, the rectangular scanning area can be interpreted as the union of three distinct subspaces (i.e., Ω, Γ_1_, and Γ_2_) as depicted in Figure 3.

Therefore, if a desired main peak lobe is inside the subspace Ω, some radiation elements must provide an element factor equal to *EF_1_* (i.e., *y_m_* = 1). Then, according to the pointing directions of the other simultaneous peak lobes, the remaining elements can provide an element factor equal to *EF_2_*, *EF_3_*_,_ or *EF_4_* (i.e., *y_m_* = 0). If all the main lobe peaks are within Γ_1_ (Γ_2_) subspace, all the radiating elements are characterized by an element factor of *EF_3_* (*EF_4_*). Conversely, in the case of the main beams involving both the Γ_1_ and Γ_2_ subspaces, or are inside the same subspace Ω, a unique element factor equal to *EF_2_* or *EF_1_* can be chosen. The boundary subspace Ω depends on the alternative *EF* shapes and could highlight the subspace within which, *EF_1_* element gain is superior to the other gains.

## 3. Performance Comparison of a 16 × 16 SMBPA

**Figure 4:**
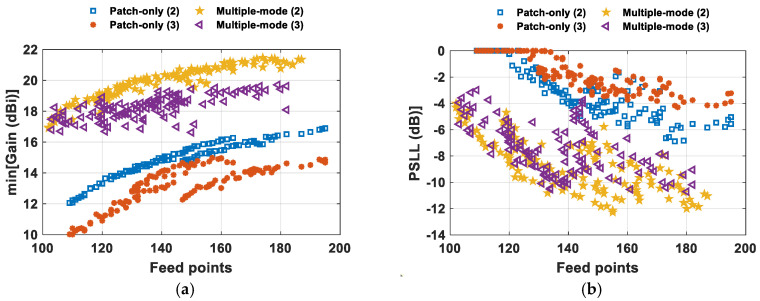
Pareto fronts comparison by considering a 16 × 16 SMBPA with two (2) and three (3) main lobe peaks; (**a**) minimum array gain and (**b**) PSLL.

The novel architecture strategy for the design of SMBPA, based on mixed, multiple, and dynamic antenna element factors and the Penrose subarray clustering technique, has been applied to a 16 *×* 16 array of elements arranged in a regular triangular lattice with an interelement spacing of 0.5 λ. A multi-objective algorithm based on the Pareto dominance concept has been used for the phased array synthesis to cope with the conflicting objectives, namely the minimization of the feed points (i.e., subarrays), the maximization of the minimum array gain along the main beam pointing directions, and the minimization of the PSLL achieved during the scan of the ten different SMB configurations. Figure 4 displays the Pareto fronts highlighting the trade-off in terms of the minimum array gain and the PSLL as a function of the number of feed points with two and three concurrent main lobe peaks. For comparison purposes, the SMBPA radiative performance is also reported in Figure 4 in the case of a single-mode antenna element factor, namely when all the radiating elements have a fixed *EF_1_*. In general, the minimum array gain value along the different SMB pointing directions (Figure 4a) gently decreases for both the phased array architectures as the employed feed points diminish with respect to the fully populated array (FPA) case (i.e., 256 feed points). However, the proposed SMBPA architecture outperforms the single-mode one thanks to the multiple antenna element factor feature. Indeed, for dual-beam pointing directions and partitions with feed points greater than 180, the two Pareto fronts differ in gain by about 4.8 dB. Decreasing the number of irregular and contiguous subarrays causes a higher and higher gain difference in favor of the SMBPA with multiple antenna element factor mode up to the point where a gain difference of more than 6 dB is achieved for 109 feed points. The improvement in the minimum gain value in the case of the multiple-mode strategy is further emphasized in the case of a triple-beam pattern. In fact, for array partitioning with more than 140 subarrays, the gain difference turns out to be around 5 dB in favor of the multiple-mode strategy and tends to be around 7 dB in the case of clustering with 110 feed points.

The minimum gain value rise turns out to be of essential importance in a max–min fairness (MMF) policy, where the maximization of the minimum of a metric of interest, such as spectral efficiency (SE) or SINR, among all users represents the main goal. Additionally, Figure 4b highlights the remarkable capability of the multiple antenna element factor mode to be effective in keeping at acceptable levels the PSLL, evaluated as the highest lateral lobe assessed in all the investigated SMB configurations inside the scanning area of Figure 3. Indeed, the feed point reduction in a single-mode architecture jeopardizes the SMB radiation capability and suffers from grating lobes onset for array partitions lower than 120 and 134 feed points with two and three main lobe peaks, respectively. Conversely, the SMBPA with multiple element factors achieves a PSLL < −10 dB by employing array partitions with at least 131 feed points for both SMB radiations with two and three main lobe peaks. The remarkable decrease in the PSLL in the case of a multiple antenna element factor notably reduced inter-beam interference, thus leading to better beam separation in multiuser and multibeam systems with a consequent improvement in SINR.

**Figure 5:**
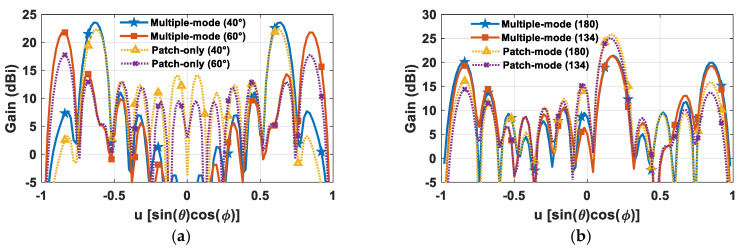
(**a**) SMBPA gain with 131 feed points and a dual-beam pointing at *θ* = ±40°, *ϕ* = 0° and *θ* = ±60°, *ϕ* = 0°; (**b**) SMBPA gain with 134 and 180 feed points with a triple-beam pointing at *θ* = ±60°, *ϕ* = 0° and *θ* = 10°, *ϕ* = 0°.

The gain of a 16 *×* 16 SMBPA has been evaluated in the case of different partition arrangements for both dual and triple-beam radiation (Figure 5).

Specifically, Figure 5a shows the dual-beam radiation in the case of 131 feed points when the two main beams point to large scanning angles, namely at *θ* = ±40°, *ϕ* = 0° and *θ* = ±60°, *ϕ* = 0°. As it can be seen, despite the considerable feed point reduction with respect to the FPA (i.e., 49% less), the SMBPA that adopts a multiple antenna element factor ensures a remarkable reduction in the scan loss as well as maintaining the lateral lobes significantly below the two main beams, thus widening the scan angle range. Conversely, the patch-only architecture is prone to superior scan loss and lateral lobes rising. Figure 5b displays an SMB with triple-beam pointing at *θ* = ±60°, *ϕ* = 0° and *θ* = 10°, *ϕ* = 0° in the case of a clustered array partition with both 134 and 180 feed points. The multiple-mode element factor strategy presents three similar peak gain values in addition to noticeably improving the minimum phased array gain, thus widening the scan angle range. Conversely, a more pronounced peak gain unbalance as well as higher lateral lobes occur when adopting a single-mode antenna element factor scheme.

**Figure 6:**
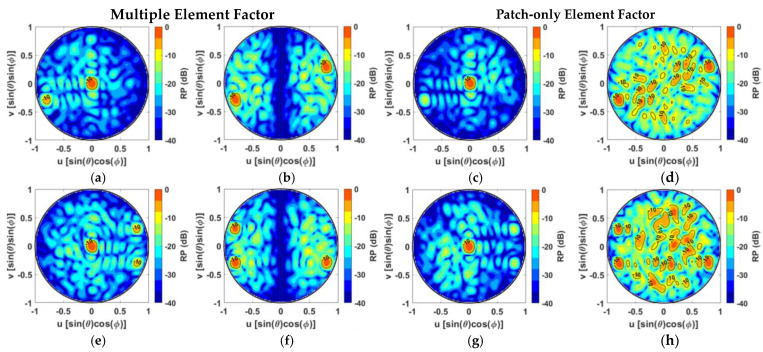
Normalized multibeam RP with two main lobe peaks for a Penrose-clustering-based 16 × 16 SMBPA with 131 feed points by exploiting (**a**,**b**) a multiple antenna element factor scheme and (**c**,**d**) a patch-only element factor scheme; 16 × 16 SMBPA with triple-beam RP with 128 feed points by exploiting (**e**,**f**) a multiple antenna element factor scheme and (**g**,**h**) a patch-only element factor scheme.

For a more comprehensive overview, the normalized RPs for two of the ten SMB configurations with two and three main lobe peaks are reported in the color maps of Figure 6. The multiple antenna elements factor scheme with 131 feed points (Figure 6a,b) focuses most of the energy toward the desired dual-beam directions by guaranteeing lateral lobes lower than −10 dB regardless of the two main lobe peaks’ pointing directions. Conversely, in the case of a patch-only element factor SMBPA solution (Figure 6c,d), a decreased ability to concentrate the radiation toward the main beam directions as well as considerable radiation spreading in the visible region (PSLL = −2.6 dB) occurs to the point that the two desired main peaks cannot be clearly identified. In the case of triple-beam radiation, despite the large scanning pointing directions and the halving of the available feed points, the architecture based on a multiple antenna element factor scheme better focuses the energy toward the desired main beam directions by producing just a few undesired lateral lobes with a PSLL of around −9 dB (Figure 6e,f). Conversely, the patch-only case is subject to severe radiation spreading over the visible region with a PSLL = −1.5 dB for the SMB configurations involving large scan angles (Figure 6h) as well as large scan losses and strong peaks gain unbalance for triple-beam radiation layouts that include a central beam (Figure 6g).

**Figure 7:**
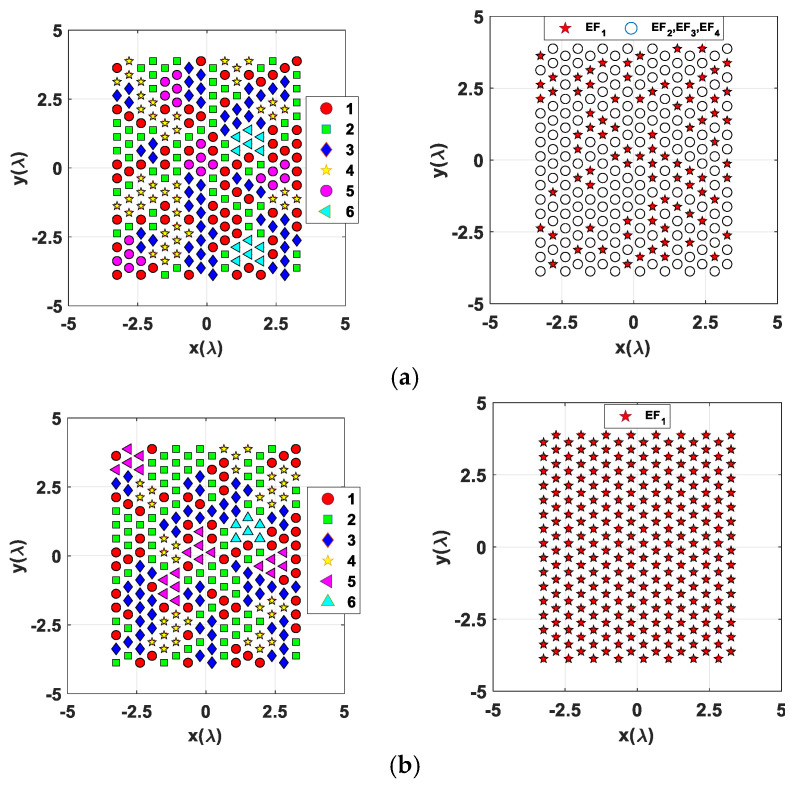
Array clustering layout (left column) and antenna element factors (right column) for a Penrose-based 16 × 16 triangular lattice SMBPA with 131 feed points and two main lobe peaks with (**a**) a multiple antenna element factor scheme and (**b**) a single-mode (*EF_1_*) antenna element factor scheme.

**Figure 8:**
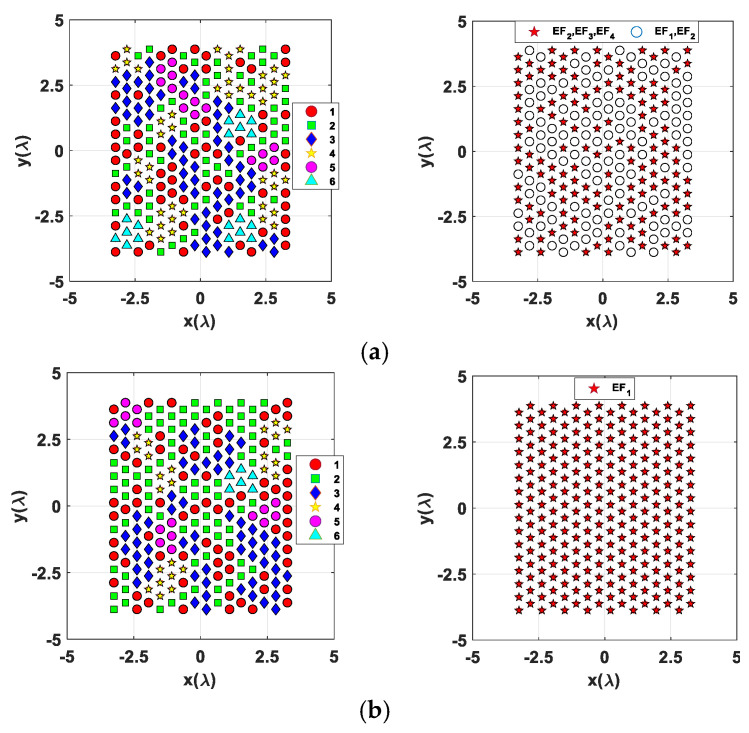
Array clustering layout (left column) and antenna element factors (right column) for a Penrose-based 16 × 16 triangular lattice SMBPA with 128 feed points and triple-beam radiation with (**a**) multiple-mode architecture and (**b**) a single-mode (*EF_1_*) architecture.

The corresponding array partition layouts comprising subarrays with up to six radiating elements and the selected antenna element factors are depicted in Figure 7 and Figure 8 for SMB configurations with two and three main lobe peaks, respectively.

The element factor layout for the multiple-mode architecture shown in Figure 7a (right column) highlights which elements of the SMBPA must select a patch-mode (*EF_1_*) element factor (red stars markers) in the cases of *Conf#2.1* and *Conf#2.2* of dual-beam configuration, and more generally when one of the two desired beams points within the subspace *Ω* shown in Figure 3. Conversely, the other SMBPA radiating elements can provide an element factor equal to *EF_2_*, *EF_3_*_,_ or *EF_4_* (Table 1). The element factor layout for the multiple-mode architecture shown in Figure 8a (right column) emphasizes the two radiating element groups that can provide an element RP equal to *EF_1_* or *EF_2_* (highlighted with white circles markers) or *EF_2_*, *EF_3_*_,_ or *EF_4_* (red stars markers according to the triple-beam configurations) in the case of triple-beam radiation as described in Table 1.

## 4. Statistical Analysis of a 16 × 16 SMBPA

To further assess the improvement guaranteed by the proposed SMBPA architecture based on multiple and dynamic element factor schemes as well as prove its robustness, different radiative configurations within the rectangular scanning area (Figure 3) have been evaluated by considering the SMBPA partition layout with 134 feed points retrieved in case of the triple-beam radiation synthesis. Specifically, different SMB pointing directions with four and five main lobe peaks have been randomly selected from uniformly distributed points within the rectangular scanning area shown in Figure 3 by assuring a minimum main beam peak distance of 10 degrees. A statistical analysis of the SMB radiative performance has been assessed by considering 1000 iterations of different SMB pointing directions.

**Figure 9:**
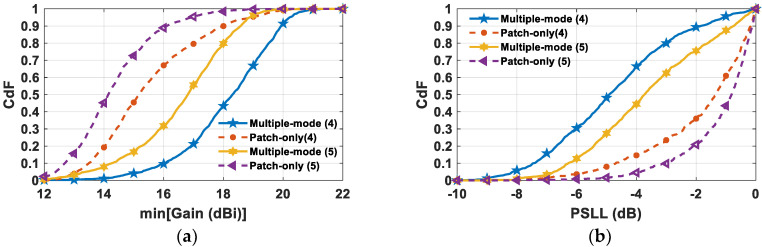
CdF of the (**a**) minimum gain and (**b**) PSLL in the case of a 16 × 16 SMBPA comprising 134 feed points and SMB pointing directions with four and five main lobe peaks.

The cumulative distribution functions (CdFs) reported in Figure 9 clearly emphasize that the multiple-mode SMBPA synthesis surpasses the patch-only architecture also from a statistical point of view. In fact, the improvements are highlighted by a better mean value (e.g., CdF = 0.5) as well as the general trend. Specifically, the proposed strategy allows an enhancement of the mean value of the minimum gain with respect to the patch-only-based SMBPA design scheme (Figure 9a) of 3.2 dBi and 2.7 dBi in the cases of SMB with four and five main lobe peaks, respectively. Moreover, the multiple-mode SMBPA strategy highlights a probability of 90% and 70% of having a minimum gain value higher than 16 dBi for SMB with four and five main lobe peaks, respectively; whereas in the single-mode architecture, this probability is only the 33% and 10%, respectively. Regarding the PSLL (Figure 9b), the multiple-mode scheme mean values are −5 dB and −3.8 dB with a probability of having PSLL > −3 dB of 20% and 35% for SMB with four and five main lobe peaks, respectively. Conversely, the patch-only SMBPA produces PSLL mean values superior to −1.3 dB with a probability of having PSLL > −3 dB of 78% and 90% for SMB with four and five main lobe peaks, respectively. Finally, these results for four and five beams are obtained from an array optimized for three main lobes, thus highlighting the flexibility and adaptability of the proposed SMBPA design.

## 5. Conclusions

A novel phased array architecture able to provide simultaneous multiple-beam patterns has been addressed. The described approach, based on the MR linear precoding weights that could be implemented with any BF method, overcomes the limitations due to the performance impairments encountered when employing a single-mode antenna element factor scheme by taking advantage of a multiple antenna element factor scheme. This feature is integrated into a Penrose tessellation strategy for the partitioning of a periodic phased array into irregular subarrays, thus reducing the TRM modules. The envisioned strategy therefore merges the benefit of using mixed and multiple-mode element factor schemes with a dynamic selection of their RPs. In fact, according to the SMB pointing directions, the individual array elements can dynamically select one of the available sets of RPs. Synthesized results based on SMB with two and three main beams inside a rectangular scanning area have emphasized that, compared with traditional phased arrays based on a single antenna element factor, the proposed novel SMBPA scheme can provide significant advantages in terms of feed points reduction and widening of the scan angle range with a more balanced peaks gain values, as well as a notable lowering of PSLL. A statistical assessment has also been provided to prove the reliability of the proposed approach even in the cases of four and five concurrent main beams. These distinct radiative improvements make the proposed antenna array architecture an attractive candidate for SMB communication scenarios.

## Figures and Tables

**Table 1:** Set of SMB Configurations Independently Scanning Five Predefined Directions and the Allowed Multiple Antenna Element Factor.

N° Beams	N° Conf	Main Beams (*θ*, *ϕ*)			Multiple Antenna Element Factors
2	Conf#2.1	0°, 0°	60°, ±20°		EF_1_, EF_4_
	Conf#2.2	0°, 0°	60°, ±160°		EF_1_, EF_3_
	Conf#2.3	60°, 20°	60°, −20°		EF_4_
	Conf#2.4	60°, 160°	60°, −160°		EF_3_
	Conf#2.5	60°, 20°	60°, ±160°		EF_2_
		60°, −20°	60°, ±160°		
3	Conf#3.1	0°, 0°	60°, 20°	60°, −20°	EF_1_, EF_4_
	Conf#3.2	0°, 0°	60°, 160°	60°, −160°	EF_1_, EF_3_
	Conf#3.3	0°, 0°	60°, 20°	60°, ±160°	EF_1_, EF_2_
			60°, −20°	60°, ±160°	
	Conf#3.4	60°, 20°	60°, 160°	60°, −160°	EF_2_
				60°, −20°	
		60°, −20°	60°, −160°	60°, 20°	
				60°, 160°	

## Data Availability

Data are contained within the article.
